# Study of lncRNA TPA in Promoting Invasion and Metastasis of Breast Cancer Mediated by TGF-β Signaling Pathway

**DOI:** 10.3389/fcell.2021.688751

**Published:** 2021-08-06

**Authors:** Qinglin Li, Wenju Mo, Yuqin Ding, Xiaowen Ding

**Affiliations:** The Cancer Hospital of the University of Chinese Academy of Sciences (Zhejiang Cancer Hospital), Institute of Basic Medicine and Cancer (IBMC), Chinese Academy of Sciences, Hangzhou, China

**Keywords:** prognostic significance, lncRNA TPA, TGF-β, metastasis, breast cancer

## Abstract

**Purpose:**

This study was to investigate the effects of lncRNA TPA overexpression and knockdown in stable transfected cell lines on the EMT, migration and invasion capabilities of breast cancer cells.

**Methods:**

WB and qRT-PCR were used to detect the expression of E-cadherin, Vimentin, fibronectin and N-cadherin, the key molecules of EMT, to determine whether lncRNA regulates EMT; scratch, migration and invasion assay were used to detected the effect of lncRNA TPA on the migration and invasion of breast cancer cells. The effect of lncRNA TPA on breast cancer metastasis was observed in nude mice model. Pierce Magnetic RNA-Protein Pull-Down Kit was used to bind the 3′-terminal desulfurized biotin-labeled lncRNA TPA with Magnetic beads, and then incubated with the proteins extracted from cell line C and D, respectively. After elution of the binding proteins, the interacting proteins were further identified by mass spectrometry to screen out the interacting proteins. The candidate proteins were expressed and purified *in vitro*, and the interaction between lncRNA-candidate proteins were verified by RNA-EMSA.

**Results:**

Overexpression of lncRNA TPA decreased the expression of E-cadherin, and significantly increased the expression of Vimentin, fibronectin and TGF-β1 (*p* < 0.01), and increased the migration rate, migration ability and invasion ability of cell group (*P* < 0.01). Multiple lung metastases were observed in the lung tissue of nude mice with overexpression of lncRNA TPA.

**Conclusion:**

LncRNA TPA affects the occurrence of breast cancer EMT through TGF-β signaling pathway, and then promotes the invasion and metastasis of breast cancer. LncRNA TPA may affect the corresponding signaling pathways through one or more interacting proteins, and ultimately promote the invasion and metastasis of breast cancer.

## Introduction

According to the latest data of American Cancer Society and China National Cancer Center, breast Cancer patients in the United States and China account for 26.8–29% of all female tumors, and the incidence of breast Cancer is on the rise (Chen et al., 2016; Siegel et al., 2016). With the progress of early prevention, early diagnosis and comprehensive treatment of breast cancer, although the mortality rate of breast cancer has not increased significantly, the distant metastasis of tumor is still the most important factor affecting the prognosis of breast cancer, and about 90% of the deaths of cancer patients are caused by tumor metastasis. How to prevent the spread and metastasis of breast cancer is a great difficulty in the treatment of breast cancer, and has always been a hot issues in breast cancer research. The key to solve this challenge is to deeply understand the specific mechanism of breast cancer metastasis and carry out precise treatment and prevention accordingly.

Tumor metastasis is a multi-step process caused by specific gene changes under internal and external environment. It is regulated by multiple factors and involves multiple genes, and needs to go through a series of continuous and selectable cascading events. Studies have shown that (Thiery and Lim, 2013; Bill and Christofori, 2015) epithelial-mesenchymal transition (EMT) plays a crucial role in the metastasis of breast cancer, which is the key initial step of tumor metastasis. EMT is mainly characterized by the transformation of polar epithelial cells into transitional mesenchymal cells. After epithelial cells are transformed into mesenchymal cells, their adhesion ability is reduced, and their migration, invasion ability and anti-apoptosis ability are significantly enhanced (Sarri et al., 2008; Thiery et al., 2009; Araki et al., 2011; Beach et al., 2011). The occurrence of EMT is the result of the coordination and co-action of many factors, usually accompanied by changes in the expression of epithelial cell-specific genes such as E-cadherin (CDH1) and interstitial cell-specific genes such as Snail and Zeb2 (Sekiya and Suzuki, 2011; Shah and Kakar, 2011; Wang D. et al., 2011; Chaw et al., 2012). E-cadherin is the most typical marker of EMT, and its down-regulation can promote the occurrence of EMT in breast cancer cells, and then promote the metastasis of breast cancer cells (Jin et al., 2014). The down-regulation factors of E-cadherin included the increased expression of Vimentin and the up-regulation of transforming growth factor-β (TGF-β). Snail is negatively correlated with the expression of E-cadherin, and its high expression in epithelial tumors can promote the migration and invasion of tumor cells, thereby causing EMT. The mechanism may be that it affects the signaling pathways of TGF-β/Smad/Zeb, NF-κB/Twist, and 3-phosphate Inositol (PI3K/Akt). Recent studies have shown that non-coding RNA also plays a pivotal role in this process. For example, several members of the miRNA-200 family (miR-200a, 200b, 200c, miR141 and miR-429, etc.) can regulate EMT by directly affecting the expression of E-cadherin and vimentin (Gregory et al., 2008; Wang Y. et al., 2011). In addition, tumor microenvironment also plays an important role in EMT, especially the cytokines of TGF-β family of transforming growth factors (Medici et al., 2011). However, so far, most studies on the regulatory mechanism of EMT are limited to genes and miRNAs, while few studies have been conducted on long non-coding RNAs (lncRNAs), especially lncRNAs related to TGF-β induction.

## Materials and Methods

### Material

#### Cell and Animal

MCF-7 were provided by Jiangsu Kaiji biological technology Co., Ltd. The complete culture medium of cell lines was 90%DMEM+10%FBS, which was cultured in the culture medium of 37°C and 5%CO_2_ saturated humidity. BALB/c nude mice (Changzhou Vince Biotechnology Development Co., ltd. Certificate. Animal production license: SCXK (Su 2011-0003), Experimental animal license: SYXK (Su) 2011-0036. The ethics institutional review board of Zhejiang Cancer Hospital approved the protocols for data collection and analyses. All the methods described here were performed in accordance with the relevant guidelines and regulations.

#### Medicines and Reagents

*β*-Actin were purchased from SIGMA Co., Ltd., MI, United States. E-cadherin and Vimentin were obtained from CST Co., Ltd., fibronectin and TGF-β1 were obtained from Abcam Co., Ltd., United States. The fluorescent quantitative PCR reagent: primers were obtained from Shanghai SANGON Biological Technology Service Co., Ltd., Shanghai, China. RNA extraction reagent TriZol was purchased from Bio RT reagent Kit Co., Ltd., Canada. PrimeScriptTM (Perfect Real Time) was supplied by Bao Biotechnology Co., Ltd., Dalian, China.

#### Methods

##### Cell culture

MCF-7 cells were cultured in RPMI-1640 medium containing 10% fetal bovine serum and placed at 37°C in a 5% CO2 incubator. The cells grew in monolayers, and the culture medium was discarded when the cells covered over 80% of the culture flask bottom. Trypsin (0.25%) was added to digest the cells for 1–2 min. When the cells became round, equal volume of serum-containing culture medium was added to terminate digestion. Cells were suspended by blowing with a pipette and transferred to a 15 ml centrifuge tube for centrifugation at 1,000 r/min for 5 min. With supernatant discarded, 1–2 ml of culture medium was added. The cells were resuspended and transferred to a new culture flask. The cells were cryopreserved by adding 1 ml of cryopreservation solution for every 5 × 10^6^ cells. Before use the cells were resuscitated and dethawed rapidly at 37°C. Cryopreservation solution was removed by centrifugation and culture medium was added. The cells adhered to the wall the next day and proliferated actively 2–3 days after inoculation. Cells reaching the log phase were harvested.

The following groups were set up: A: Normal breast cancer cells; B: lncRNA TPA overexpression cells; C: lncRNA TPA knockdown cells.

#### Establishment of Animal Model

BALB/C female nude mice, 4 weeks old, provided by Shanghai slick company, animal production license No. scxk (Shanghai) 2012-0021. Eighteen nude mice were taken. The logarithmic growth phase breast cancer cells were collected, and the cell concentration was adjusted to 1 × 10^7^ cells/ml. The nude mice were subcutaneously inoculated with 0.2 ml on the right side of the neck, and then kept in SPF environment for 2–3 weeks. The animals were randomly divided into three groups when the tumor proliferated to palpable time. The growth of tumor was observed and measured every 3 days. After 4 weeks, the mice were killed to measure the size and weight of tumor.

### Immunofluorescence Detection of the Effect of lncRNA TPA Overexpression and Knockdown on the Expression of E-Cadherin, Fibronectin, TGF-β1 and Vimentin Antibodies in MCF-7 Cells

Immunohistochemical methods were used to detect the expression of E-cadherin, Vimentin, fibronectin and N-cadherin in normal breast cancer cells, lncRNA TPA overexpressing stable transfected breast cancer cell lines, and knockdown stable transfected breast cancer cell lines.

1) Put a sterile high-cleanliness cover glass (intercellular cabinet, small glass slide) into the six-well plate, and then plant the six-well plate at a cell density of 60–70%, and the cells will adhere to the wall overnight;
2) Add 1–2 ml of pre-cooled 4% paraformaldehyde (PFA), after fixation for 10 min, rinse with PBS three times, 5 min each time;
3) Add 1–2 ml 0.2% Triton X-100 to each well, treat for about 2 min, rinse with PBS three times, 5 min each time;
4) Add 80 μl of the four primary antibodies (diluted with 5% goat serum according to the instructions) on each cover glass (diluted with 5% goat serum according to the instructions), and incubate at 4°C overnight;
5) Aspirate PBS, add 80 μl secondary antibody to each cover glass (diluted with 5% goat serum according to the instructions, usually 1:500), incubate at room temperature for 0.5 h, and aspirate the secondary antibody;
6) Add 2 ml PBS to each well to rinse;
7) Aspirate PBS and add nuclear stain reagent DAPI to each well for staining for 2 min.
8) Aspirate the nucleus staining reagent, rinse with PBS three times, then add 1 ml PBS to keep it moist;
9) First add an appropriate amount of anti-fluorescence quenching mounting media to the slide glass, use a fine needle to lift the cover glass, press the cell side down onto the slide glass, from one side of the cover glass to the other Press it over and suck up the excess mounting media with absorbent paper.
10) Observe and take pictures under a fluorescence microscope. Image J software calculates the fluorescence intensity.

### The Effect of lncRNA TPA Overexpression and Knockdown on Cell Migration

Take normal breast cancer cells (MCF-7) in logarithmic growth phase, overexpress lncRNA TPA and knock down stable transfected breast cancer cells, count, adjust the concentration, and use cell scratches to detect cell migration ability.

### The Effect of lncRNA TPA Overexpression and Knockdown on Cell Invasion

Dilute Matrigel with serum-free RPMI 1640 culture medium at a ratio of 3:1, take 30 μl of Matrigel dilution, evenly coat the Transwell chamber, and incubate overnight at 4°C; put the Transwell chamber into a 24-well culture plate, and add 200 μl (5 × 10^4^ cells) of the above three cell suspensions, add 500 μl of RPMI1640 culture medium containing 10% fetal bovine serum to the lower chamber. According to the above group, place it in a 37°C, 5% CO2 incubator for 24 h, wipe off the matrigel and the bottom cells of the upper chamber with a cotton swab, fix with 4% paraformaldehyde for 10 min, wash three times with PBS, and stain with 0.1% crystal violet staining solution for 30 min. Then take pictures and count the number of cells invaded into the lower chamber; the experiment was repeated three times. Transwell was used to detect the invasion ability of lncRNA TPA overexpression and knockdown stable cell lines.

### RNA-Pull Down and Mass Spectrometry Identification

#### *In vitro* Transcription

##### Reaction System Ratio (20 μl)

1) Seal, mix, and incubate at 37°C for 40 min

2) Remove DNA template and free nucleotides
3) Add 28 μl denuclease water to make the volume to 50 μl
4) Add 5 μl 5 M ammonium acetate, vortex to mix
5) Add 60 μl absolute ethanol
6) Centrifuge at high speed for 10 min, discard the supernatant, rinse once with 70% ethanol, add an appropriate amount (15–20 μl) of nucleic acid-free water to dissolve, and divide into 5 μl/tube for use

### RNA Pull-Down Experiment

Use the RNA pull-down kit to do RNA pull-down experiments to adsorb proteins that interact with RNA. The method is as follows.

1) Probe labeling of target RNA

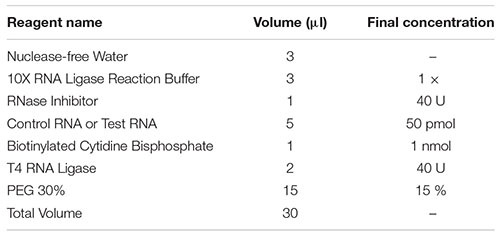

2) The labeled target RNA is bound to streptavidin magnetic beads

1) Add 30 μl of streptavidin magnetic beads to a 1.5 ml centrifuge tube.
2) Place the above 1.5 ml centrifuge tube on the magnetic stand, collect the magnetic beads, and discard the liquid.
3) Add an equal volume of 1X RNA Capture Buffer. Resuspend the magnetic beads and gently blow.
4) Add 50 mol biotin-labeled RNA and mix well.
5) Incubate at room temperature for 15–30 min.

3) Protein binds to labeled RNA

1) Place the above 1.5 ml centrifuge tube on the magnetic stand, collect the magnetic beads, and discard the liquid.
2) Dilute 10X Protein-RNA Binding Buffer to 1X (that is, add 10 μl Protein-RNA Binding Buffer to 90 μl ultrapure water).
3) Add 100 μl 1X Protein-RNA Binding Buffer and mix well.
4) The main components of the protein binding reaction of the prepared RNA (Table 1).
5) Place the above 1.5 ml centrifuge tube on the magnetic stand, collect the magnetic beads, and discard the liquid.
6) Combine 100 μl of the mixed RNA protein binding reaction solution with the magnetic beads. Resuspend the magnetic beads and gently blow.

4) Elution of RNA-binding protein complexes

1) Put the above 1.5 ml centrifuge tube on the magnetic stand, collect the magnetic beads, take out the supernatant, and save it for analysis.
2) Add 50 μl Elution Buffer and vortex with magnetic beads to mix. Incubate at room temperature for 15 min.
3) Collect the eluate and save at −80.

**TABLE 1:** The main components of the protein binding reaction of the prepared RNA.

**Reagent**	**Volume of each component (per 100 μ L) M l**	**Range**
10X Protein-RNA Binding Buffer	10	5–20 μL
50% glycerin	30	0–50 μL
Lysis solution (protein concentration >2 mg/ml)	20–30	200 μg or more
Nuclease-free ultrapure water	Up to 100	To 100 μL
Total capacity	100	

### Protein Profile Identification

1) Protein reductive alkylation and enzymatic hydrolysis
The reductive alkylation of the protein is as follows: add the final concentration of 10 mM dithiothreitol (DTT) to reduce the protein, then add the final concentration of 55 mM iodoacetamide (IAM), and finally add 1 μg Trypsin enzyme, overnight enzymatic hydrolysis for 8–16 h.
2) Treatment after enzymolysis
The peptides produced by enzymatic hydrolysis are desalted on a C18 column. After the desalted peptides are drained, the peptides are dissolved in 15 μl Loading Buffer (0.1% formic acid, 3% acetonitrile).
3) LC-MS/MS Identification of Protein Enzymatically Hydrolyzed Peptides

The peptides were analyzed by LC-MS/MS (ekspert nanoLC; AB Sciex TripleTOF 5600-plus) instrument, and then the results were evaluated.

#### qPCR Detection

The primer sequence information used in qRT-PCR is shown in Table 2.

**TABLE 2:** Primer sequence.

**Gene**	**Forward Primer**	**Reverse Primer**
Human ACTG	GGGAACAAAAGGCGGGGTC	ATGGAAGGAAACACGGCTCG
Human ENOA	CGGGAATCCCACTGTTGAGG	CCATGGGCTGTGGGTTCTAA
Human BIP	GAACGTCTGATTGGCGATGC	ACCACCTTGAACGGCAAGAA
Human G3P	AATGGGCAGCCGTTAGGAAA	GCCCAATACGACCAAATCAGAG
Human ALDOA	GGGCTCCCTCCCCATCAATA	TAGGGAAACCTGAAGCCCCT
Human TBB5	ATCCAGAGCAGGGAAAGCTG	CTCAGGCCGTTGTTCTAGGG
Human lncRNA TPA	TGAAACTGCTCGGGCTGAAT	GAACGTACCCCACACAGGAG

### RNA Extraction

Sample processing: Add 1,000 μl of Trizol to the homogenization tube for every 200 mg of tissue, and place the lysed sample at room temperature for 5–10 min to completely separate the nucleoprotein and nucleic acid. Place in an ultra-clean table, incubate at room temperature for 5 min, and centrifuge at 12,000 rpm for 10 min. Aspirate the supernatant into a new 1.5 mL centrifuge tube, add 200 μl of chloroform, shake well, let stand at room temperature for 2 min, 4°C, 12,000 rpm, and centrifuge for 10 min. Aspirate the supernatant to a new 1.5 mL centrifuge tube, add 600 μl of isopropanol, mix well, let stand at room temperature for 15 min, 4°C, 12,000 rpm, centrifuge for 15 min, discard the supernatant. Add 1 ml of 75% absolute ethanol (750 μl of absolute ethanol and 250 μl of DEPC water) to rinse the pellet, centrifuge at 12,000 rpm at 4°C for 5 min, and discard the supernatant. Add 1 mL of absolute ethanol, rinse the pellet, centrifuge at 12,000 rpm at 4°C for 5 min, discard the supernatant, and dry at room temperature for 10 min. Add 40 μl of DEPC water to dissolve RNA and store in a refrigerator at −80°C for later use.

### Reverse Transcription Reaction

Prepare the following reaction system for reverse transcription reaction. Reaction conditions: 42°C, 15 min; 85°C, 5 min.


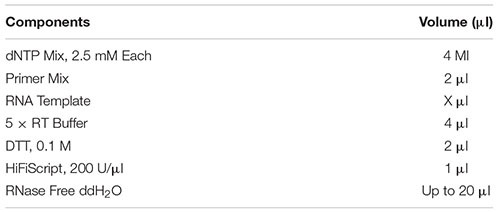

1) Prepare the following reaction system for real-time fluorescent quantitative PCR reaction. Mix the solution in the tube thoroughly with a vortex shaker, and centrifuge briefly at low speed.

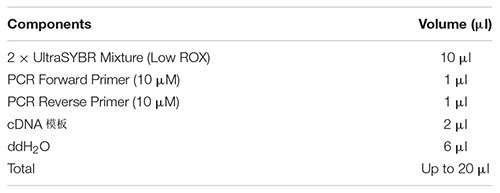

2) Spotting: Add the mixed liquid in step (1) to the well plate, and ensure three replicate wells for each gene in each sample.
3) PCR reaction: The PCR program is optimized, and the 8-tube plate that has been sampled in step (2) is placed on the Realtime PCR machine for PCR reaction.
4) Reaction conditions: 95°C, 10 min denaturation; 95°C, 15 s; 60°C, 60 s; 40 cycles.

#### Statistical Analysis

Results were reported as mean ± SD, and analyzed statistically using SPSS 19.0 software. Intergroup comparisons were carried out by One-way ANOVA, and *P* < 0.05 was considered significant.

## Results

### The Effect of lncRNA TPA Overexpression and Knockdown on the Protein Expression of E-Cadherin, Vimentin, Fibronectin, and TGF-β1

**FIGURE 1:**
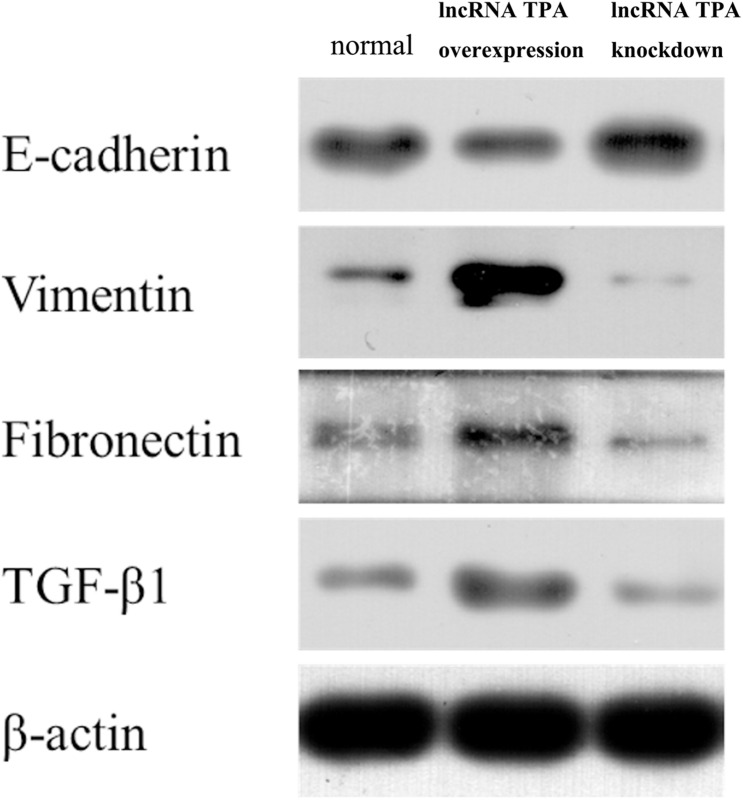
The protein expression levels of E-cadherin, Vimentin, fibronectin, and TGF-β1 in stably transfected breast cancer cell lines with lncRNA TPA overexpression and knockdown ($\bar{\chi }$ ± s, *n* = 3).

**FIGURE 2:**
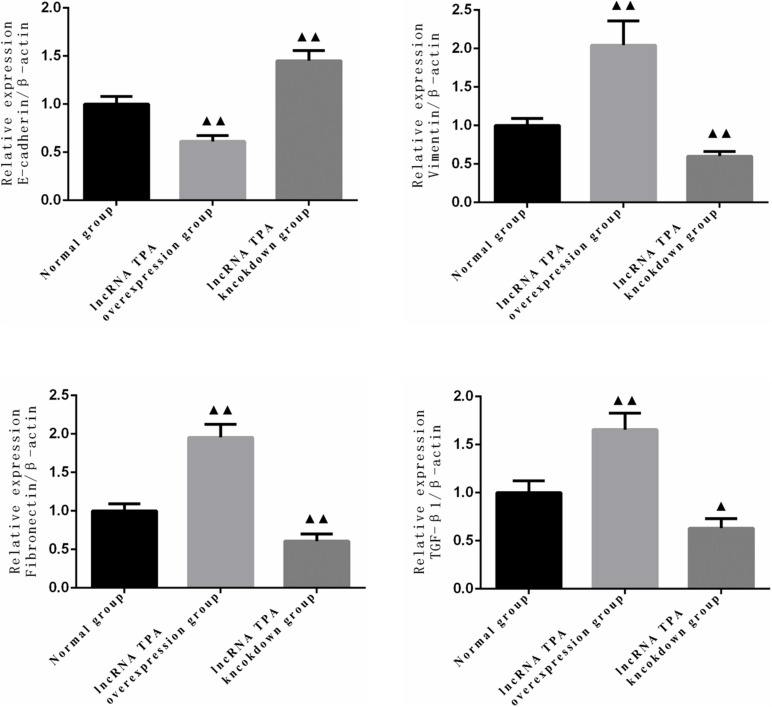
Statistics of protein expression levels of E-cadherin, Vimentin, fibronectin and TGF-β1 in lncRNA TPA overexpression and knockdown stably transfected breast cancer cell lines (x ± s, *n* = 3). ▲▲Indicating *p* < 0.01 compared with the control group, ▲Indicating *p* < 0.05 compared with the control group.

It can be seen from Figures 1, 2 that compared with the normal breast cancer cells, the expression of E-cadherin protein in the lncRNA TPA overexpression cells was significantly reduced (*p* < 0.01), and the protein expression of Vimentin, fibronectin and TGF-β1 were significantly increased (*p* < 0.01). Compared with the normal breast cancer cells, the expression of E-cadherin protein in the lncRNA TPA knockdown cells was significantly increased (*p* < 0.01), and the protein expression of Vimentin, fibronectin and TGF-β1 was significantly reduced (*p* < 0.01).

### Immunofluorescence Detection of the Effect of lncRNA TPA Overexpression and Knockdown on Antibody Expression

**FIGURE 3:**
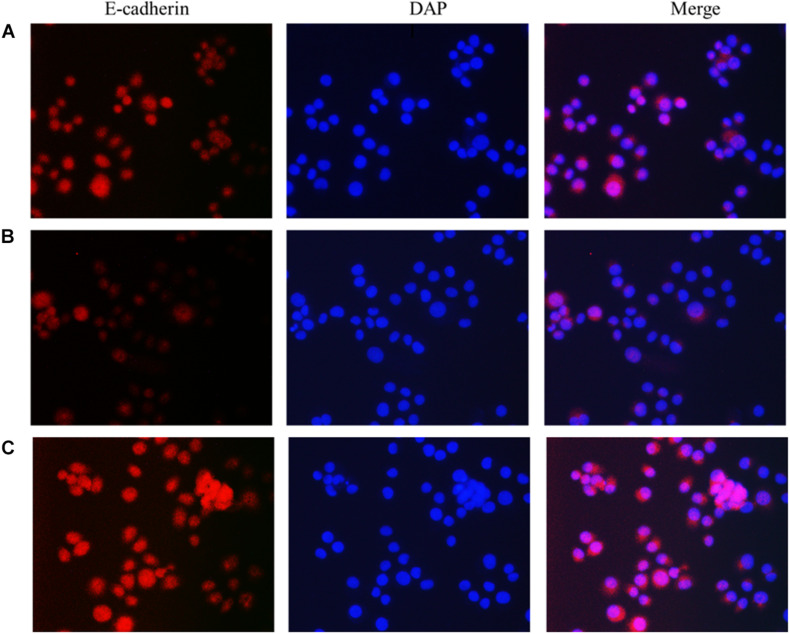
The effect of lncRNA TPA overexpression and knockdown on the expression of MCF-7 E-cadherin antibody [200×, **(A)** Normal breast cancer cells, **(B)** lncRNA TPA overexpression cells, **(C)** lncRNA TPA knockdown cells].

**FIGURE 4:**
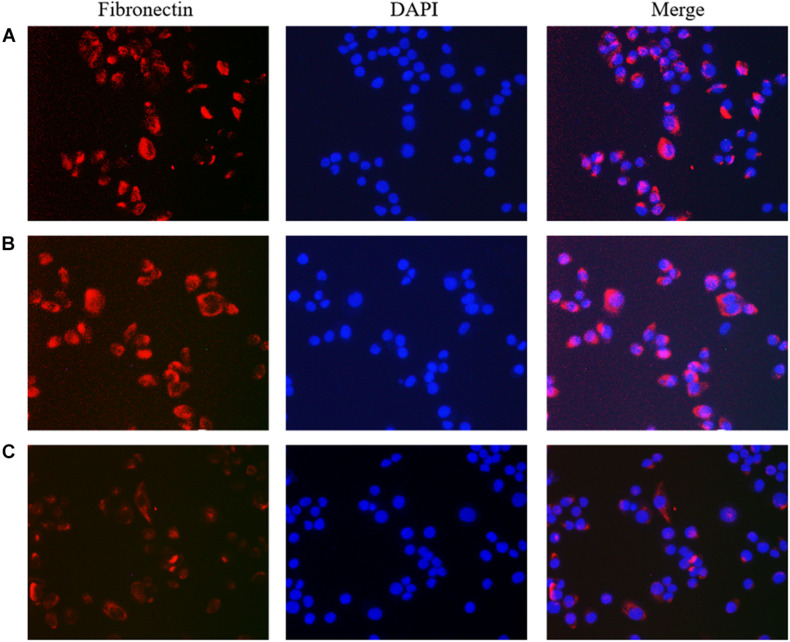
The effect of lncRNA TPA overexpression and knockdown on the expression of MCF-7 Fibronectin antibody [200×, **(A)** Normal breast cancer cells, **(B)** lncRNA TPA overexpression cells, **(C)** lncRNA TPA knockdown cells].

**FIGURE 5:**
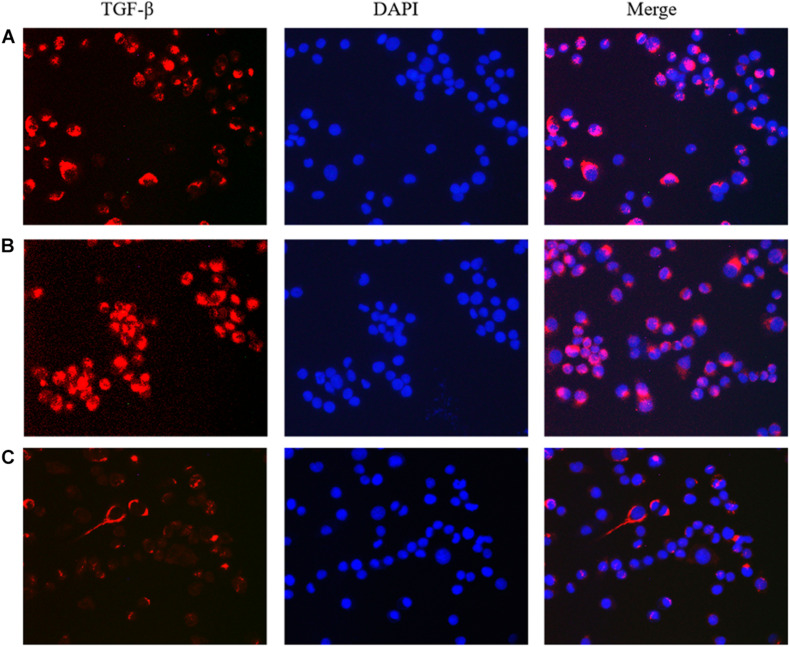
The effect of lncRNA TPA overexpression and knockdown on the expression of MCF-7 TGF-β antibody [200×, **(A)** Normal breast cancer cells, **(B)** lncRNA TPA overexpression cells, **(C)** lncRNA TPA knockdown cells].

**FIGURE 6:**
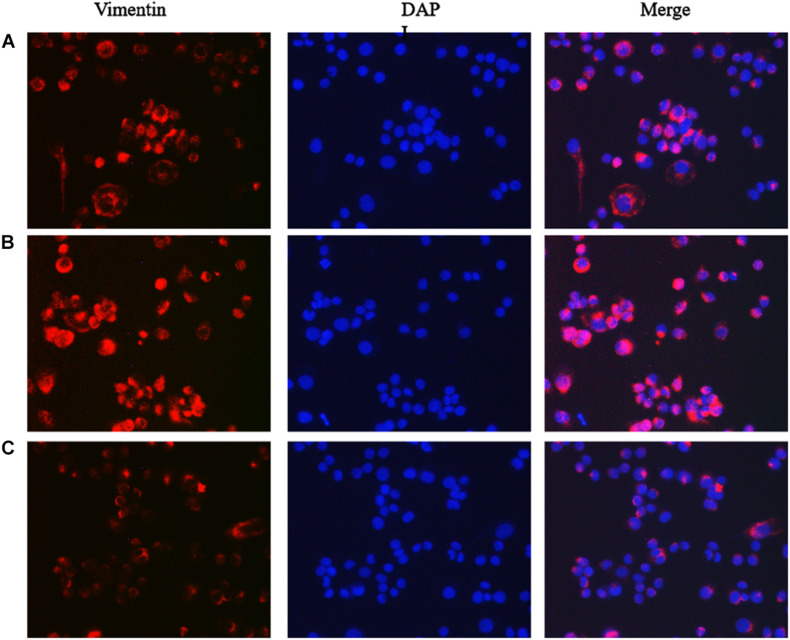
The effect of lncRNA TPA overexpression and knockdown on MCF-7 Vimentin protein expression [200×, **(A)** Normal breast cancer cells, **(B)** lncRNA TPA overexpression cells, **(C)** lncRNA TPA knockdown cells].

The results showed that compared with the normal breast cancer cells, the expression of E-cadherin in the lncRNA TPA overexpression cells was reduced, and the expression of Fibronectin, TGF-β1 and Vimentin were significantly increased. The E-cadherin expression in the lncRNA TPA knockdown cells was significantly increased. The cadherin increased, the expression of Fibronectin, TGF-β1 and Vimentin were all significantly decreased, and the differences was statistically significant (*P* < 0.05, *P* < 0.01). Among them, the four proteins of E-cadherin, Fibronectin, TGF-β1 and Vimentin are mainly expressed in the cytoplasm and cell membrane. The results are shown in Figures 3–6 and Table 3.

**TABLE 3:** The effect of lncRNA TPA overexpression and knockdown on the expression of MCF-7 Vimentin antibody ($\bar{\chi }$ ± S, *n* = 3).

**Groups**	**Immunofluorescence intensity of MCF-7 protein expression**			
	**E-cadherin**	**Fibronectin**	**TGF-β**	**Vimentin**
Normal breast cancer cells	56.13 ± 8.60	46.77 ± 3.55	38.45 ± 6.34	39.91 ± 2.49
lncRNA TPA overexpression cells	32.13 ± 7.45*	65.03 ± 3.40**	55.2 ± 3.93*	54.77 ± 3.25**
lncRNA TPA knockdown cells	72.52 ± 5.16*	28.17 ± 4.61**	28.89 ± 7.29**	31.64 ± 5.87**

Compared with the normal group *p < 0.05;**p < 0.01.

### The Effect of lncRNA TPA Overexpression and Knockdown on the Migration Ability of MCF-7 Cells

**FIGURE 7:**
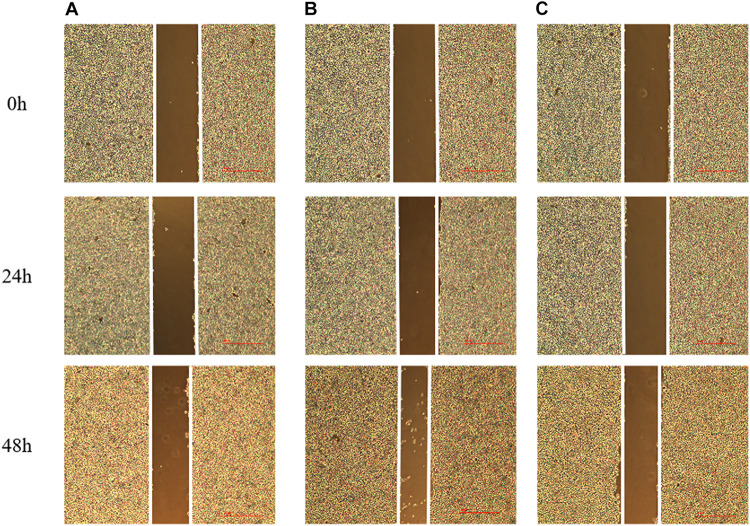
The effect of lncRNA TPA overexpression and knockdown on the migration rate of MCF-7 cells [40×, **(A)** Normal breast cancer cells, **(B)** lncRNA TPA overexpression cells, **(C)** lncRNA TPA knockdown cells].

Compared with the normal breast cancer cell group, the migration rate of the lncRNA TPA overexpression cells was significantly increased, and the migration rate of the lncRNA TPA knockdown cells was significantly reduced after the scratches were performed for 24 and 48 h, respectively (*p* < 0.01, *p* < 0.01); The results are shown in Figure 7 and Table 4. Compared with the normal group, the lncRNA TPA overexpression group has the strongest migration ability, and the lncRNA TPA knockdown group has the weakest migration ability.

**TABLE 4:** The effect of lncRNA TPA overexpression and knockdown on cell migration ($\bar{\chi }$ ± S, *n* = 3).

**Groups**	**Cell migration rate (%)**	
	**24 h**	**48 h**
Normal breast cancer cells	8.00 ± 1.15	14.06 ± 0.65
lncRNA TPA overexpression cells	12.04 ± 0.88**	20.48 ± 0.99**
lncRNA TPA knockdown cells	4.20 ± 0.55**	11.17 ± 0.76**

Compared with the normal group *p < 0.05; **p < 0.01.

### The Effect of lncRNA TPA Overexpression and Knockdown on the Invasion Ability of MCF-7 Cells

**FIGURE 8:**
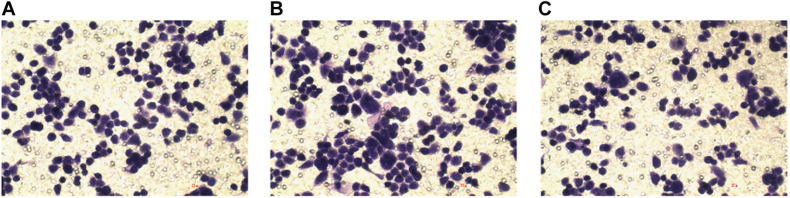
The effect of lncRNA TPA overexpression and knockdown on cell invasion [200×, **(A)** Normal breast cancer cells, **(B)** lncRNA TPA overexpression cells, **(C)** lncRNA TPA knockdown cells].

Compared with the normal breast cancer cell group, the cell invasion ability of the lncRNA TPA overexpression group was significantly increased, while the cell invasion ability of the knockdown group was significantly reduced, and the difference was statistically significant (*P* < 0.01). The results are shown in Figure 8 and Table 5.

**TABLE 5:** The effect of lncRNA TPA overexpression and knockdown on cell invasion (*upchi* ± S, *n* = 3).

**Groups**	**Normal breast cancer cells**	**lncRNA TPA overexpression cells**	**lncRNA TPA knockdown cells**
Cell invasion number	199.33 ± 8.08	233.33 ± 10.02*	173.00 ± 7.00*

Compared with the normal group *p < 0.05; **p < 0.01.

### HE Staining to Observe the Pathological Changes of Lung Tissue in Nude Mice With Breast Cancer

**FIGURE 9:**
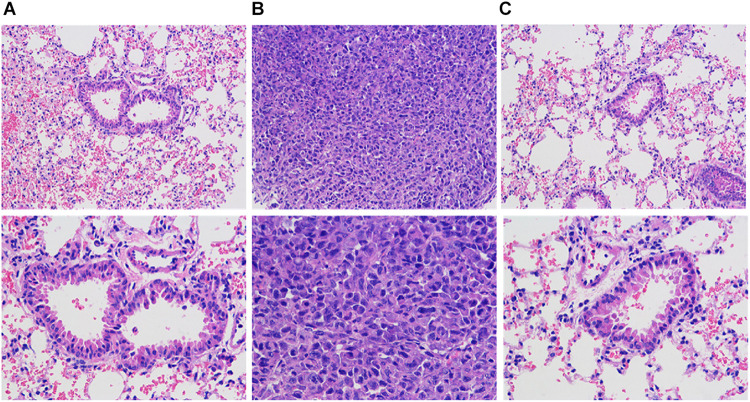
HE staining to observe the pathological changes of lung and lung metastases in nude mice with breast cancer (HE staining, 200X). **(A)** Normal breast cancer cells, **(B)** InCRINA TPA overexpression cells, **(C)** IncRNA TPA knockdown cells.

Figure 9 is a lung tissue section of nude mice with breast cancer. In group B overexpression cells, multiple lung metastases can be observed on the lung tissue of nude mice. The sample site is lung metastasis. It can be seen that the morphology and structure of lung metastases are similar to tumor tissues. Can be judged as breast cancer metastasis. As low-metastasis group cells, no metastases were found in lung tissues in groups A and C. The sample site was lung tissue. It can be seen that there is a large amount of inflammatory cell infiltration in the lung tissue of tumor nude mice, and some alveoli have alveolar wall thickening or incomplete alveolar structure, etc., variety.

### RNA-Pull Down and Mass Spectrometry Results

#### *In vitro* Transcription of RNA

##### PCR product amplification to obtain DNA template

Design PCR primers for sense chain and antisense chain. The primer sequence is shown in the following Table:


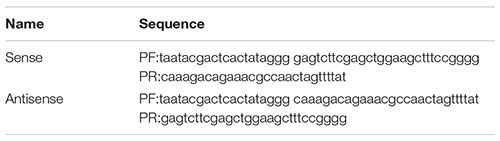



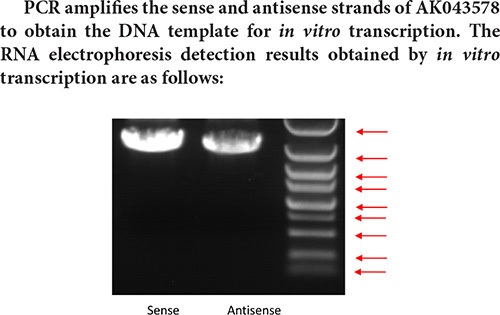


Marker strip size from top to bottom is: 5 k 3 k 2 k 1.5 k 1 k 750 bp 500 bp 250 bp 100 bp.


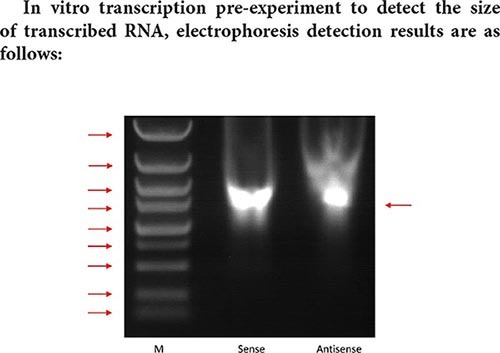


Marker strip size from top to bottom is: 5 k 3 k 2 k 1.5 k 1 k 750 bp 500 bp 250 bp 100 bp.

**Result analysis:**

Judging from the electrophoresis detection diagram of the *in vitro* transcription pre-experiment, the main band size is about 1.6 k, and the full-length sequence of AK043578 cannot be transcribed. Therefore, the pull-down experiment cannot be performed normally, so the transcribed sequence is directly used for interaction protein screening.


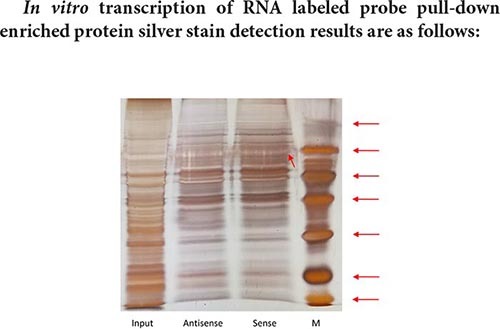


The size of the Marker strip from top to bottom is: 116KD 66.2KD 45KD 35KD 25KD 18.8KD 14.5KD.

**Result analysis:**

The result of pull-down enriched protein detection with a segment of RNA that has been transcribed is as shown in the silver staining diagram above. From the silver staining diagram, it can be seen that there are more binding proteins, but the difference bands visible to the naked eye are not very obvious. It may be that the amount of differential protein enrichment is small, and mass spectrometry is needed to further confirm the specific differential protein.

### Protein Profile Identification

#### Proteinpilot Search

After the LC-MS/MS is off the machine, the original off-machine data is directly submitted to the Proteinpilot software connected to the AB SCIEX Triple TOF^TM^ 5600 plus mass spectrometer for database search, please refer to Table 6.

**TABLE 6:** Proteinpilot search parameters.

**Item**	**Value**
Type of search	Identification
Enzyme	Trypsin
Cys Alkylation	Iodoacetamide
Instrument	Triple TOF 5600
Bias Correction	TRUE
Background Correction	TRUE
ID focus	Biological modifications
Search Effort	Thorough ID
Protein Mass	Unrestricted
Database	uniprot_swissprot_Homosapiens_contaminants (Total 20412 protein sequences)

### Appraisal Result Statistics

In this experiment, when the confidence level of conf ≥ 95% and Unique peptides ≥ 1 is set, the number of secondary spectra generated by the sample′s mass spectrum are 15,523 and 16,229, and the number of analyzed secondary spectra are 1,037 and 959, respectively. Filter out common contaminated proteins and peptides that can be matched with them. For the total number of peptides and proteins identified in each sample, please refer to Table 7.

**TABLE 7:** Protein identification by LC-MS/MS.

**Sample name**	**Total number of spectra**	Number of identification spectra*	**Spectrum resolution rate (%)**	Number of identified peptides*	**Identify the number of proteins**	Unique-2**
AK04_ antisense	15523	1037	6.68	291	80	50
AK04_ sense	16229	959	5.91	281	101	47

### Comparison Between Protein Samples

There are two samples in this experiment. The proteins identified in each sample are not only different in quantity, but also different proteins may exist in different samples or the same protein may exist in different samples at the same time.

The figure below shows the Venn diagram of the differential protein collection between the experimental sample AK04_sense-AK04_antisense. It can be seen from the figure that a total of 115 proteins were identified, of which 66 proteins were simultaneously identified in two samples, and the unique proteins identified by AK04_sense and AK04_antisense, respectively. The numbers are 35 and 14, please refer to Venn_Result for related result information.

### Protein Related Information

Simply analyze the information related to the mass spectrometry results of the identified proteins. When the confidence level is conf ≥ 95% and Unique peptides ≥ 1, after filtering out common contaminating proteins, the total number of proteins identified in the AK04_antisense and AK04_sense protein samples are 80 and 101, respectively. Some of the protein-related information with the top scores are shown in Table 8. Among them, for the coverage of the target protein sequence information, in the sequence coverage pane of the Proteinpilot software, the sequence is displayed in green and its credibility is above 95%, and the credibility of the yellow is 50∼95% (can be Reference), the credibility of red is 0–50% (uncredible), and the ones shown in gray can be considered completely unreliable and completely ignored. But if the sequence is at the -COOH end, even if it is gray, it is not necessarily wrong, because -COOH is easily filtered out by LC-MS/MS. For information about all other proteins and Subsets proteins, see Table ProteinSummary.xlsx.

**TABLE 8:** Protein Related Information Sheet.

**Protein ID**	**Coverage (%)**	**Mass (Da)**	**Unique Peptide**	**Identified by**
sp| P63261| ACTG_HUMAN	49.0700006484985	41792.5	22	
sp| P06733| ENOA_HUMAN	35.9400004148483	47168.6	14	
sp| P11021| BIP_HUMAN	22.7799996733665	72332.4	10	AK04_antisense
sp| P04406| G3P_HUMAN	28.659999370575	36053.0	11	
sp| P04075| ALDOA_HUMAN	20.3299999237061	39419.7	12	
sp| P63261| ACTG_HUMAN	40.0000005960464	41792.5	1	
sp| P04075| ALDOA_HUMAN	25.8199989795685	39419.7	11	
sp| P11021| BIP_HUMAN	18.0399999022484	72332.4	9	AK04_sense
sp| P07437| TBB5_HUMAN	22.3000004887581	49670.5	3	
sp| P06733| ENOA_HUMAN	25.3500014543533	47168.6	10	


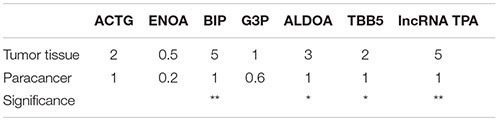


#### qPCR Test Results

**FIGURE 10:**
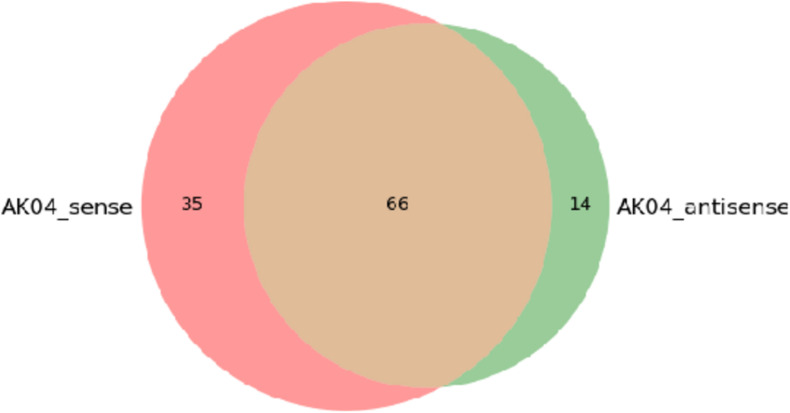
AK04 sense-AK04 antisense Venn diagram between two samples.

**FIGURE 11:**
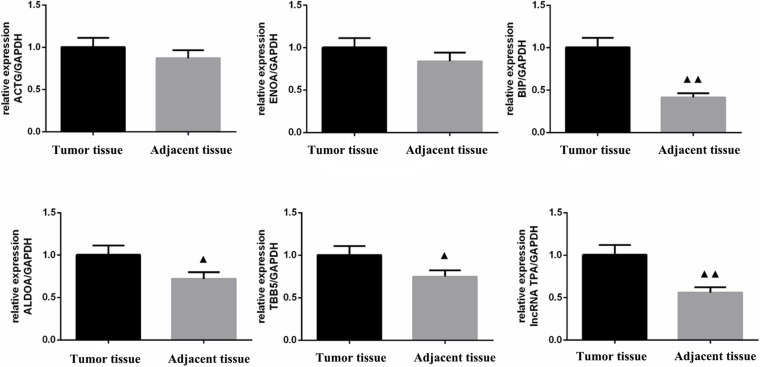
ACTG, ENOA, BIP, ALDOA, TBB5, and lncRNA TPA mRNA expression levels in breast cancer tissues (χ ± s, *n* = 3). Compared with the control group, ▲*P* < 0.05; ▲▲*P* < 0.01.

According to Table 9 and Figures 10, 11 compared with the tumor tissue group, the variety of the expression levels of ACTG and ENOA in the paracancerous group was not significant (*P* > 0.05), and the expression levels of BIP, ALDOA, TBB5 and lncRNA TPA were significantly reduced (*P* < 0.05 or *P* < 0.01).

**TABLE 9:** ACTG, ENOA, BIP, ALDOA, TBB5 and lncRNA TPA mRNA expression levels in breast cancer tissues (−$\bar{\chi }$ ± *s*, *n* = 3).

	**ACTG**	**ENOA**	**BIP**	**ALDOA**	**TBB5**	**lncRNA TPA**
Tumor tissue	1.00 ± 0.11	1.00 ± 0.11	1.00 ± 0.11	1.00 ± 0.11	1.00 ± 0.10	1.00 ± 0.12
Paracancer	0.87 ± 0.09	0.84 ± 0.10	0.42 ± 0.05^▲▲^	0.72 ± 0.08^▲^	0.75 ± 0.07^▲^	0.56 ± 0.06^▲▲^

*Compared with the control group, ^▲^*P* < 0.05,^▲▲^*P* < 0.01.*

## Discussion

Long chain length of the non-coding RNA (lncRNA) is a kind of more than 200 nt, which does not have ability of functional RNA molecule coding protein, can be in epigenetic, transcription and the transcription level raised or lowered the expression of target genes, involved in chromatin remodeling and transcription interference, alternative splicing, and many other physiological and pathological process, exert the function of oncogenes or tumor suppressor genes, thus affecting the tumor invasion and metastasis (Lander et al., 2001; Alexander et al., 2010). The changes of lncRNA content and expression level in breast cancer tissues are often correlated with the occurrence of metastasis and the prognosis of patients. A study of more than 900 breast cancer samples showed that 215 lncRNAs were abnormally expressed in breast cancer tissue compared to normal breast tissue. Luminal A-specific lncRNAs are associated with PI3K (phosphatidylinositol 3-kinase), FGF (fibroblast growth factor), and activation of TGF-β pathways. Basal like specific lncRNAs are related to EGFR (epidermal growth factor receptor-dependent signaling pathway) and EMT (Van Grembergen et al., 2016). For example, H19 gene (Berteaux et al., 2005), LOC554202 (Aμgoff et al., 2012), as well as their host genes miR-31 and steroid receptor RNA activator (SRA) (Novikova et al., 2012; Beato and Vicent, 2013) can affect the occurrence, development and metastasis of breast cancer from different links. In addition, a limited number of studies have shown that lncRNA can affect the EMT process of breast cancer cells. For example, Hox antisense intergenic RNA (HOTAIR) (Rinn et al., 2007; Gupta et al., 2010; Zhang et al., 2014) of Hox gene is often significantly up-regulated in primary and metastatic breast cancer, which increases the ability of tumor cell invasion and metastasis and is closely related to prognosis. It can also regulate the EMT of breast cancer stem cells by indirectly inhibiting miR-7 and then affecting the STAT3 signaling pathway. Metastasis-associated transcriptome 1(MALAT-1) of lung adenocarcinoma (Gutschner et al., 2013; Xu et al., 2015), whose abnormal expression can not only affect the invasion and metastasis of breast cancer cells, but also affect the EMT of breast cancer cells by regulating PI3K-Akt signaling pathway. Downregulation of lncRNA CCAT2 can inhibit the proliferation and invasion of breast cancer cells by regulating the TGF-β pathway and promoting the apoptosis of breast cancer cells (Wu et al., 2017). It can be seen that abnormal expression of lncRNA is closely related to the activation, EMT and metastasis of TGF-β pathway in breast cancer (Wang et al., 2016). However, although our mechanism of breast cancer metastasis and lncRNA in which have a certain understanding of the role of, but ultimately solve the problem is still a long way to go, the identification of functional lncRNA is only the tip of the iceberg, is still an urgent need to explore new lncRNA in breast cancer metastasis, and clarify its regulatory mechanism, eventually service in clinical.

Therefore, we screened the candidate lncRNA AK043578 from TGF-β-induced mouse NMuMG cells by using lncRNA microarray technology, confirming that lncRNA AK043578 can regulate the EMT of NMuMG and promote cell invasion. The expression level of AK043578 in allogeneic mouse breast cancer cells with different metastatic potential is also different, suggesting that the candidate lncRNA AK043578 plays an important role in the EMT of tumor and the invasion and metastasis of breast cancer. Furthermore, we identified that the human homologous analog AK043578(h) (named lncRNA TPA), which is differentially expressed in different human breast cancer cell lines. Therefore, we assumed that lncRNA TPA was closely related to breast cancer EMT and metastasis. The mechanisms involved may include as follows: 1. LncRNA TPA affects the occurrence of breast cancer EMT through the TGF-β signaling pathway, thereby promoting the invasion and metastasis of breast cancer; 2. LncRNA TPA may affect the corresponding signaling pathways through one or more interacting proteins, and ultimately promote the invasion and metastasis of breast cancer. And these are exactly what this subject tries to verify and answer. Therefore, this project intends to analyze and verify the function and molecular mechanism of TGF-β-induced novel breast cancer metastasis-related lncRNA TPA through overexpression and RNA interference, Western blot, RNA pull down experiment, and mass spectrometry identification, aiming to clarify the role and specific mechanism of lncRNA TPA in breast cancer EMT, invasion and metastasis. Further study on its clinical application value will hopefully become a new molecular marker and drug therapeutic target for breast cancer metastasis, providing new opportunities and new opinion for accurate diagnosis, precise treatment and precise prevention of breast cancer metastasis.

## Conclusion

LncRNA TPA affects the occurrence of breast cancer EMT through TGF-β signaling pathway, and then promotes the invasion and metastasis of breast cancer. LncRNA TPA may affect the corresponding signaling pathways through one or more interacting proteins, and ultimately promote the invasion and metastasis of breast cancer.

## Data Availability Statement

The data presented in the study are deposited in the article/supplementary material, repository, accession number 20210233.

## Ethics Statement

All animal testing programs were approved by the Zhejiang University Laboratory Animal Management Committee for review and approval. The ethics institutional review board of Zhejiang Cancer Hospital approved the protocols for data collection and analyses. All the methods described here were performed in accordance with the relevant guidelines and regulations.

## Author Contributions

QL and XD conceived and designed the experiments. WM and YD performed the experiments. WM analyzed the data. QL wrote the manuscript. XD revised the manuscript. All authors have reviewed the final manuscript.

## Conflict of Interest

The authors declare that the research was conducted in the absence of any commercial or financial relationships that could be construed as a potential conflict of interest.

## Publisher’s Note

All claims expressed in this article are solely those of the authors and do not necessarily represent those of their affiliated organizations, or those of the publisher, the editors and the reviewers. Any product that may be evaluated in this article, or claim that may be made by its manufacturer, is not guaranteed or endorsed by the publisher.
